# Facilitation and Dominance in a Schooling Predator: Foraging Behavior of Florida Pompano, *Trachinotus carolinus*

**DOI:** 10.1371/journal.pone.0130095

**Published:** 2015-06-11

**Authors:** Meagan N. Schrandt, Sean P. Powers

**Affiliations:** 1 Department of Marine Sciences, University of South Alabama, Mobile, Alabama, United States of America; 2 Dauphin Island Sea Lab, Dauphin Island, Alabama, United States of America; UC Santa Cruz Department of Ecology and Evolutionary Biology, UNITED STATES

## Abstract

Presumably an individual’s risk of predation is reduced by group membership and this ‘safety in numbers’ concept has been readily applied to investigations of schooling prey; however, foraging in groups may also be beneficial. We tested the hypothesis that, when feeding in groups, foraging of a coastal fish (Florida Pompano, *Trachinotus carolinus*) on a benthic prey source would be facilitated (i.e. fish feeding in groups will consume more prey items). Although this question has been addressed for other fish species, it has not been previously addressed for Florida Pompano, a fish known to exhibit schooling behavior and that is used for aquaculture, where understanding the feeding ecology is important for healthy and efficient grow-out. In this experiment, juvenile Florida Pompano were offered a fixed number of coquina clams (*Donax* spp.) for one hour either in a group or as individuals. The following day they were tested in the opposite configuration. Fish in groups achieved greater consumption (average of 26 clams consumed by the entire group) than the individuals comprising the group (average of 14 clams consumed [sum of clams consumed by all individuals of the group]). Fish in groups also had fewer unsuccessful foraging attempts (2.75 compared to 4.75 hr^-1^) and tended to have a shorter latency until the first feeding activity. Our results suggest fish in groups were more comfortable feeding and more successful in their feeding attempts. Interestingly, the consumption benefit of group foraging was not shared by all – not all fish within a group consumed equal numbers of clams. Taken together, the results support our hypothesis that foraging in a group provides facilitation, but the short-term benefits are not equally shared by all individuals.

## Introduction

Many fish species form groups at some time during their life history and group behavior serves a variety of functions in different systems. Fish groups are termed ‘shoals’ when fish are loosely organized and ‘schools’ when coordinated swimming occurs [1]. In teleosts, schooling behavior is dictated by two main keys: predators and food [2–4]. A balance between the two seems to be maintained by schooling fish, but it can shift depending on prey distribution, suggesting that predator defense mechanisms do not necessarily take precedence over feeding (e.g. [5]). But, predator defense is the well-hypothesized function of schools, stemming from concepts related to safety in numbers [6, 7], the dilution effect (e.g. [8]), and heightened predator surveillance [7, 9, 10]. Nevertheless, several advantages may be conferred by group foraging.

Fish in groups have been shown to increase search efficiency (reduced search times) and allocate more time to feeding (e.g. [9]), exhibit sampling behaviors (i.e. sampling food patches of different quality [11]), alter feeding strategies to maximize energy efficiency (e.g. [12]), hunt collaboratively for mobile prey [13], and engage in passive information transfer and forage area copying behaviors (reviewed by [9]). Previous research has examined feeding responses in relation to schooling behaviors in a variety of fish species, with many results suggesting increased foraging success in groups (e.g. three-spined sticklebacks [10], Australian salmon [14], walleye Pollock [15]). However, similar studies have not been applied to Florida Pompano when feeding on a natural benthic prey source; most previous Florida Pompano group-feeding experiments have been in regards to evaluating feeding efficiency for commercial aquaculture. If indeed group membership promotes foraging success, then schooling by fish predators, like Florida Pompano, may be one of several mechanisms used to cope with difficult foraging situations. Sandy beach environments, where Florida Pompano are regularly observed, could be considered one such complex foraging habitat. At the interface of sea, land, and air, sandy beach slopes are a stressful environment—few systems compare in terms of physical stability or biological structure [16]. Consequently, prey organisms inhabiting beach slopes have a generally high mobility and the ability to burrow rapidly [16]. Intuitively, fish that feed on these organisms must then in turn have developed mechanisms to successfully forage on mobile, burrowing prey.

Many foraging strategies of schooling fish focus on the location of prey [14], and this is especially important when schooling predators are foraging on mobile aggregations that may only be briefly available [15]. However, this concept may also apply to other predator-prey relationships. The Florida Pompano (*Trachinotus carolinus*) is a fast-swimming schooling predator found in the beach surf zone. Juvenile Florida Pompano will “surf” up the beach slope in shallow water to capture prey items [17], many of which are coquina clams (*Donax* spp.) [18]. Coquina clams exhibit a unique behavior called ‘swash-riding’ wherein the clams emerge from the sediment and ride beach waves in synchrony with the tides [19]. During exposure, Florida Pompano will forage on coquinas. In this sense, coquina clams are both mobile and only briefly available when moved by wave activity, because Florida Pompano will not dig for clams once they have burrowed [17]. Although only briefly available, the clams’ emergence from the sediment and presence in the water column may be predicted by local wave activity.

Multiple theories of foraging facilitation by schooling fish are based on the patchy distribution of prey items (e.g. ephemeral prey schools). Because both replicating the natural behavior of coquina clams in the surf zone and conducting manipulative experiments in the surf zone is difficult, we approached the theory of group foraging facilitation differently. Here, we assessed whether the previous conclusions of foraging facilitation still hold with a natural prey item that is presented more uniformly and is present throughout the experimental trial (e.g. not a pulse of prey). In this sense, we assessed foraging behavior after a prey patch had been located. Coquina clams, frequently seen in large aggregations, can be one of the dominant fauna on exposed sandy beaches [20]. Therefore, once a coquina clam prey patch is located by a fish predator, the clams may potentially be perceived as an abundant and more uniformly distributed food supply. Our mesocosm experiment is also different from previous work because we used a paired test design wherein behaviors of fish foraging alone could be compared with their complementary behaviors in a group. Specifically, we hypothesized that foraging by juvenile Florida Pompano would be *facilitated* in groups (i.e. fish feeding in groups would consume more prey items).

## Materials and Methods

### Predator and prey species

Juvenile Florida Pompano (*Trachinotus carolinus*), 17–25 cm TL (total length), were the predator species used in experimental trials; they were obtained from Claude Peteet Mariculture Center (Gulf Shores, Alabama, USA), which had obtained the fish from Proaquatix (Vero Beach, Florida, USA) when they were 0.33 g (ca. 2.54–3.18 cm TL). At the mariculture center the fish were used in nutritional studies examining the effects of the number of feedings per day with pellet food and diet supplements. Fish were transported to the Dauphin Island Sea Lab (DISL, Dauphin Island, Alabama, USA) in August 2013 and were housed in an outdoor flow through mesocosm tank (diameter = 2.36 m; water depth ca. 0.6 m) for 3 months before experiments commenced. All fish remained in this tank until the night prior to their first use in a trial and were returned to this tank following trials. Florida Pompano were fed once daily a mixed diet of cut fish and squid, live coquina clams, and pellet food.

The prey species, coquina clams (Donax spp.), were collected in November 2013 from multiple local beaches near Dauphin Island, AL. Multiple beaches were necessary to obtain enough prey items for both experimental purposes and general husbandry feeding. In AL, no sampling or collection permits are required for non-managed invertebrates; therefore, coquina collections were in accordance with the laws of the state of AL. Clams were collected from the swash zone of Gulf-facing beaches using a mole crab rake (also known as a triangle sand flea rake). Coquina clams represent a significant component (up to 58%) of wild pompano diets in the area [21]. Clams were sorted so only individuals 1.2–1.6 cm in length (anterior to posterior, mean = 1.5 ± 0.002 cm) were used in experimental assays. Additionally, clams with epibiotic hydroids present were not used since Manning and Lindquist [17] reported that Florida Pompano select against clams with hydroids.

### Feeding experiments

Experiments were conducted in a set of three, recirculating indoor mesocosm tanks, unregulated for temperature (18.6 ± 0.27°C), but regulated for salinity (22.9 ± 0.15psu). Water was pumped in from Mobile Bay, AL and filtered. All trials were conducted within 8 days to minimize differences in water parameters. A fourth indoor tank was used as an overnight holding tank for the three fish involved in trials on any given day. All indoor tanks were 1.1 m in diameter with approximately 0.35 m water depth. No sand was placed on the bottom of the tanks because sand would allow the clams to bury and the Florida Pompano would not feed. All fish were fed 24 hr prior to trials.

**Fig 1:**
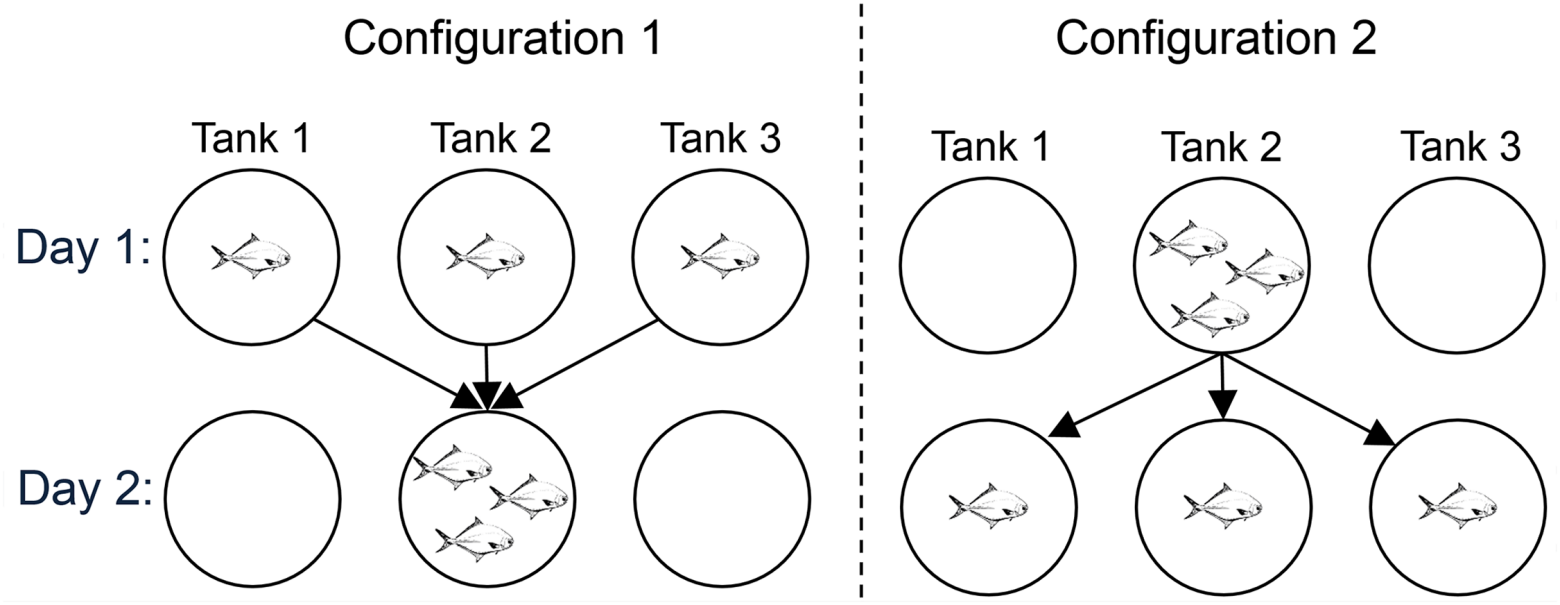
Schematic of Florida Pompano feeding trials used to test group-foraging benefits. A schematic of the two different configurations used to examine the potential for benefits of group-foraging by Florida Pompano (*Trachinotus carolinus*) feeding on coquina clams (*Donax* spp.)

The day prior to an assay, three fish were randomly selected from the outdoor flow through mesocosm tank and relocated to the indoor holding tank where lights were kept to a 12 hr light-dark cycle and fish could acclimate ca. 12 hr. The following morning, the fish were randomly tested either as individuals or as a school. Before the start of a trial, the number of tanks necessary for assays (three for individuals or one for a school) was stocked with 60 coquina clams, haphazardly placed in the tank. Preliminary trials indicated pompano would eat up to 10 clams in 1 hr so 60 clams was chosen to equate to approximately 50% of clams being consumed when 3 fish were present. Pompano were then moved to the appropriate tank(s), allowed to forage freely, and the trial was run for 1 hour. After 1 hr, the fish were removed and placed back into the overnight holding tank. The remaining coquina clams were collected from the experimental tanks and counted. The experiment was repeated the following morning with the same fish placed in the opposite configuration (Fig 1). For example, if the fish were tested as a school on day 1 then they were tested as individuals in their own tanks on day 2. After each group of fish was tested together and individually, they were measured and tagged (to ensure fish were not used again) and returned to the outdoor flow through tank. This experimental procedure was repeated four times.

All experimental trials were conducted between 0730 and 0900 because Florida Pompano feed during daylight hours [22]. Trials were recorded with a GoPro Hero2 or Hero3 camera mounted 15 cm above the center of the tank. To assess recovery efficiency of clams, we performed three trials in which a known number of clams was stocked and then recovered after an hour—recovery of clams was 100%. This experiment was conducted in accordance with animal care protocol #638305 and approved by the Institutional Animal Care and Use Committee at the University of South Alabama. Efforts were made to minimize stress and suffering in animal housing and experimental conditions.

### Data analysis

Because our hypothesis was directional (facilitation), a one-tailed paired t-test was used to compare the following three foraging actions: (1) the number of successful foraging attempts (the number of clams consumed), (2) the number of unsuccessful foraging attempts (the number of clams crushed, but not consumed), and (3) the number of attacks (the number of times fish picked up a clam and then rejected it, without consuming or crushing). The data presented here are for the minimum number of attacks, as some fish were observed to take in and reject clams repeatedly. Therefore, we have underestimated the number of attacks but the underestimation is likely similar for both individuals and schools. The number of successful and unsuccessful foraging attempts, as well as the number of attacks, for fish in groups was compared to the sum of the respective activity for the three individuals comprising the group. Observations from video data allowed for analyses pertaining to the timing of feeding activities as well as the activities of each individual within a group. A one-tailed paired t-test was used to compare the time until the first feeding event between individuals and groups. For groups, the time to first feeding was considered the time elapsed between the start of the trial (when all fish were in the experimental tank) and the first feeding activity (i.e. a fish picked up a clam). For individuals, the average time to first feeding among the three fish was calculated. If any given fish did not eat during a trial, a time of 60 min was assigned as the latency to first feeding event. Lastly, to determine whether all fish within a group foraged equally, a chi-square analysis was used to compare the observed number of clams consumed to the expected number of clams for each individual.

## Results

Feeding attempts by juvenile Florida Pompano resulted in one of three ultimate outcomes after the clam was taken into the mouth of the fish: (1) consumption of the clam (= successful foraging attempt), (2) crushing the clam but not consuming it (= unsuccessful foraging attempt), and (3) rejection of the clam without crushing the clam (= attack); Florida Pompano frequently took a clam into their mouth and rejected it, either very rapidly (almost immediately) or after several seconds.

**Fig 2:**
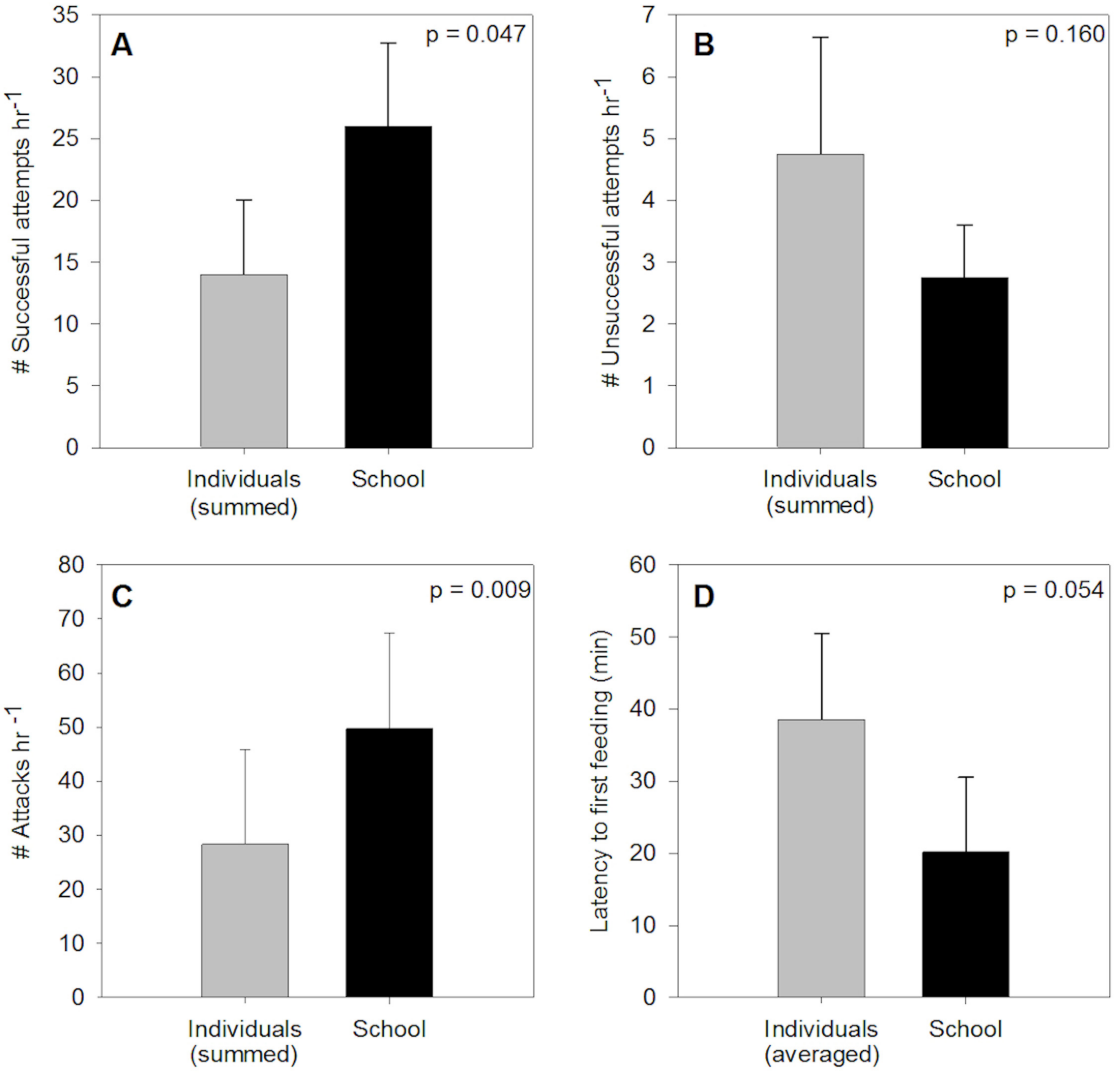
Group-foraging provides facilitation in Florida Pompano, a schooling predator, feeding on a benthic prey source. The results of the one-tailed paired t-tests for juvenile Florida Pompano foraging on coquina clams alone or in groups of three, comparing (A) the number of successful foraging attempts (= the number of clams consumed), (B) the number of unsuccessful foraging attempts (= the number of clams left crushed), (C) the number of attacks (= the number of times clams were picked up and then rejected without being crushed or consumed), and (D) the time until first feeding activity. Data are presented as mean ± SEM.

The number of clams consumed by juvenile Florida Pompano foraging in groups of three fish was greater than the sum of clams consumed by the individuals comprising their respective group (t = 2.41; p = 0.047) (Fig 2A). Consumption in groups was approximately twice the consumption of all individuals (26 vs. 14 clams). Fish foraging in groups tended to have fewer unsuccessful foraging attempts (t = -1.19; p = 0.16) (Fig 2B) and performed more attacks than fish feeding alone (t = -7.49; p = 0.009) (Fig 2C). Individual fish tended to allow more time to elapse before engaging in their first feeding activity (t = -2.79; p = 0.054) (Fig 2D).

**Fig 3:**
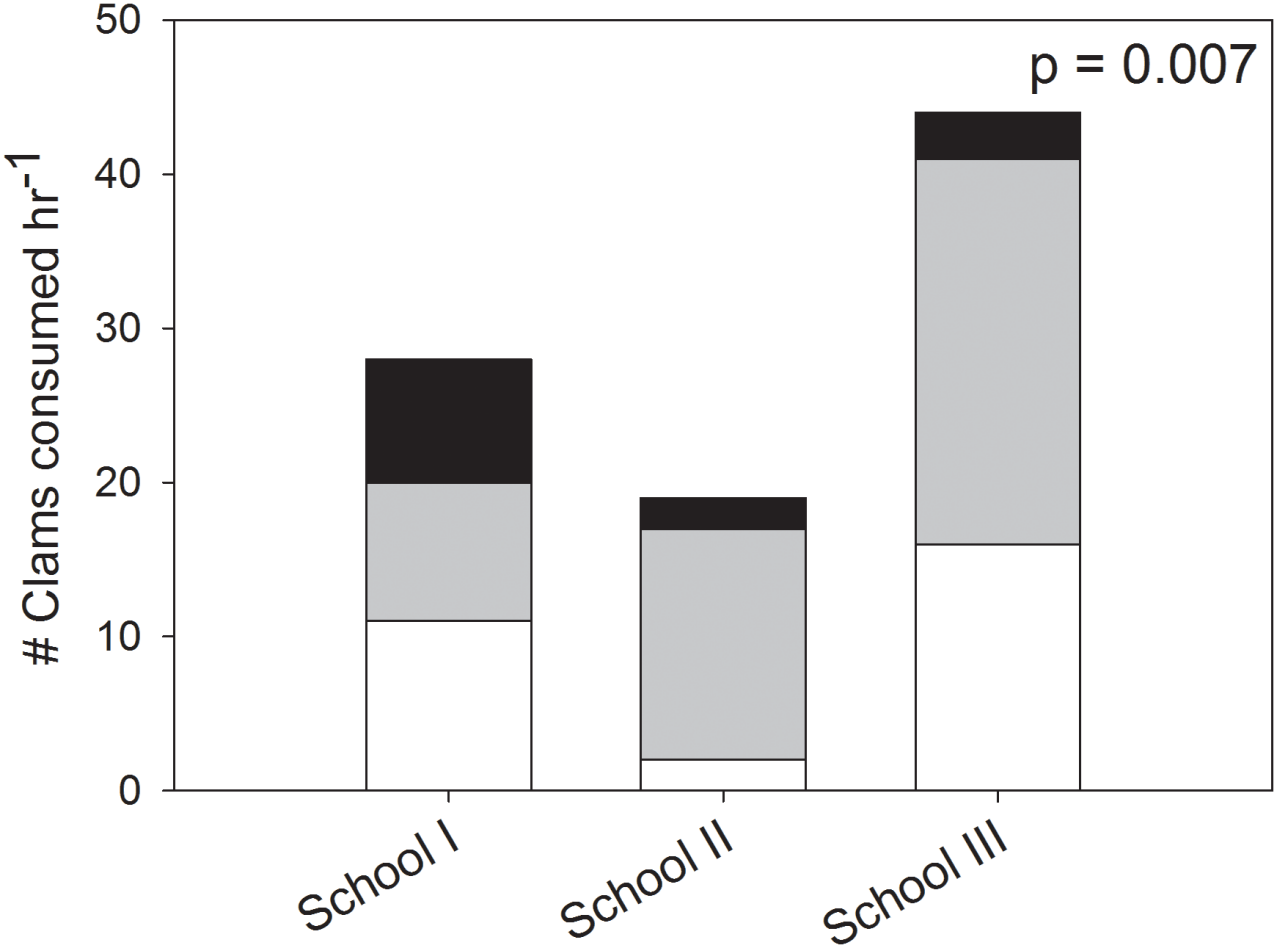
Dominance effects are present when Florida Pompano are group-foraging. A comparison, by Chi-square analysis, of the number of clams consumed by each Florida Pompano within a group. Each color within a bar represents an individual fish within the group.

For the three groups of fish with complete 1 hr videos to accompany the trial, foraging was not equal among all fish within the group (χ^2^ = 14.13; p = 0.007) (Fig 3). One group ate fairly equally (8–11 clams per fish), another had one fish that consumed the majority of the clams in the trial (15 clams) while the other two consumed very few (2 clams each), and the third group was a hierarchy—one fish ate 25 clams, another ate 16 clams, and the third fish ate 3 clams.

## Discussion

### Foraging facilitation in groups

Juvenile fish foraging in groups were more successful than those foraging alone, and also seemed to be more comfortable engaging in feeding activities. Herein, we consider ‘comfort’ to be a relative measure, meaning that fish were observed to have less of a startle response, and generally consumed and/or attempted to consume more prey items. Groups of juvenile Florida Pompano consumed more clams, performed more attacks, and tended to leave fewer crushed clams behind. This suggests juvenile Florida Pompano are both more comfortable and successful foraging in groups—the greater number of attacks (even though the clams were not ultimately consumed) while foraging in groups is likely a result of increased foraging activity—fish in groups are more likely to engage in foraging activities. Leaving fewer crushed clams behind while in groups suggests fish are more likely to take the time to complete the feeding event as opposed to picking up the prey item and then rejecting it.

Our result of increased foraging in groups is consistent with many other teleost studies (e.g., [10, 14, 15]). Increased foraging may also be the result of allocating more time to feeding when in groups e.g., [23]) and appears to be correlated with the number of individuals in a group—greater foraging success and more time allocated to feeding have been reported as group size increases (e.g., [10, 14, 23, 24]). Foraging facilitation may also occur among species, as reported by Pereira et al. [25], wherein bucktooth parrotfish were observed to take advantage of nearby sailor’s grunt schools to feed inside the highly defended territory of damselfish, thereby facilitating access to a food resource that would not normally be accessible to the parrotfish alone. But, note that other studies have reported opposite results, with less foraging success or lower feeding rates when in groups and greater foraging as solitary individuals. Furthermore, sometimes the foraging patterns are different among fish life stages. For example, four species of *Haemulon* adults were observed to have higher foraging rates when solitary as opposed to when schooling on a Brazilian coral reef [26]; but juveniles of these species did not have a clear foraging pattern between solitary and schooling individuals. Likewise, observed feeding patterns differed among life stages of *Haemulon flavolineatum* in mangroves and seagrass beds, wherein sub-adults showed no pattern between schooling and solitary individuals, large solitary juveniles spent most of their time foraging while schooling ones mainly rested, and small juveniles in seagrass beds mainly foraged when schooling [27]. In summary, patterns of fish foraging behavior in regards to schooling vs. solitary individuals can differ among species and among size or age-classes within species. Our experimental results indicated that juvenile Florida Pompano foraging in schools were more successful than those foraging individually.

In this experiment, our results suggest increased feeding in groups is a true response, and not a learned response. The fish used in experimental trials were naïve—no experimental fish were previously used for preliminary work. Juvenile Florida Pompano were also only used twice, each time for only an hour, minimizing the time available to ‘learn’ in experimental conditions. Furthermore, individual fish did not always consume more clams the second time they were in the experimental tank, regardless of whether they were tested in a group or as individuals first. Therefore, we do not believe that results were impacted by fish learning.

Additionally, we are aware that the group size (3 fish) in this experiment was small and may be a caveat to some conclusions, but we believe the results are still robust and potentially applicable to Florida Pompano populations in this area of the north-central Gulf of Mexico. Although we used the minimum school size, the Florida Pompano used in these experiments did show schooling behavior with just 3 fish in the tanks. Furthermore, Florida Pompano in this region of the Gulf coast are typically not captured in large schools (as is the case in some areas of Florida). Indeed, catch-per-unit-effort of Florida Pompano never exceeded 5 fish hr^-1^ in two years of standardized gillnet surveys (M. Schrandt, unpublished data). Lastly, we had to consider the size of the experimental tanks to be sure that Florida Pompano were not crowded and that the number of coquina clams placed in the tanks did not get so large as to nearly cover the bottom of the tank, thus not reflecting natural local coquina abundances as well. If a larger school size was used, however, we would predict that foraging success would increase with group size as has been reported previously (e.g., [10, 14, 23, 24])—to an extent. At some point, we would expect the number of fish to exceed the food resources, resulting in competition that may ultimately lead to a plateau or a decrease in the number of prey items consumed by some individuals (*see* discussion *below on individual behavioral variation and the dominance effect*).

We also observed a trend toward greater latency until feeding for fish feeding alone. We speculate the trend would have been statistically significant if one group had not waited 40 min until feeding—all other groups began feeding activity within 20 min, averaging 12.5 min. This is in opposition to individuals, who waited approximately twice as long as groups to initiate feeding activities. Engaging in feeding activities earlier when in groups is similar to previous group-foraging experiments (e.g. [9, 10, 28]). The shorter time until feeding, combined with greater consumption and less crushing, further supports our conclusion that juvenile Florida Pompano are more comfortable and successful foraging in groups.

Various hypotheses, varying in mechanism, apply to an overall increase in feeding rates in larger groups, as was observed in this experiment. These hypotheses include intragroup competition, social facilitation, and vigilance sharing (reviewed by e.g., [29]). Intragroup competition forces individuals to feed quicker. Social facilitation suggests that the willingness of any individual to feed increases with the number of individuals feeding in the group. The vigilance sharing hypothesis allows for individuals within the group to share the time spent being alert for predators, increasing the time allowed for feeding. Our data are consistent with an overall facilitation of foraging but they do not suggest any particular mechanism. It is likely that greater foraging success observed in juvenile Florida Pompano in this experiment is due to a combination of multiple explanatory hypotheses, potentially intragroup competition and/or social facilitation since a predator of Florida Pompano was not included in the experiment.

Lastly, our results indicate that juvenile Florida Pompano of similar size do not equally benefit from group-foraging. Intraspecific behavioral variation was observed during this experiment. There is a growing literature on individual variation in fish behavior, seemingly spanning all observable behaviors (e.g. activity, aggressiveness, shyness, boldness, exploration, avoidance, spawning, sociability) and the concept of behavioral syndromes in fishes is becoming more widespread (see review by [30]). For example, adult individuals of the yellow saddle goatfish *Parupeneus cyclostomus* may live solitarily (associated with searching for hidden, immobile prey items) or in groups where they exhibit collaborative hunting, with individuals performing different roles, to capture mobile prey items in corals [13]. Strübin et al. [13] examined the goatfish in their natural coral reef habitat, but here we were observing Florida Pompano in experimental tanks, where the escape response of the prey item was effectively removed—coquina clams were not able to bury into sediment. We do not believe the Florida Pompano were collaboratively hunting during this experiment because prey items were readily available on the bottom of the tank. Contrarily, the Florida Pompano may have been affected by social facilitation and/or intragroup competition (as mentioned above) to yield unequal foraging rates. It is important to consider whether fish are in competition with conspecifics because if so, activity levels (and hence, feeding) may depend on dominance rank [30].

In this experiment we observed an overall dominance effect where foraging in groups ranged from equal foraging among group members to a distinct hierarchy. Our results are similar to Milinski [31], who reported differing competitive abilities in sticklebacks (dominant fish ate 2–3 times more than subordinate fish) although they were presumed to be similar for experimental purposes. Whether this is a short- or long-term phenomenon is not known. We did not test the groups multiple times so we could not determine if the dominant fish in one trial remained dominant in subsequent trials. It is likely that this is a long-term effect of group foraging because dominance behavior resembles a positive feedback loop—dominance behavior will increase the feeding rate of dominant members and simultaneously reduce that of subordinate members [29], further increasing the disparity between dominant and subordinate individuals. Our results support the positive feedback loop characteristic because when dominance was present, the dominant individual(s) ate ≥ 2 times that of subordinate individuals.

### Maricultured vs. wild-caught fish

We exercise caution with our interpretation because we used maricultured Florida Pompano. These fish were raised under hatchery conditions and were not held in isolation prior to arriving at our facility. Despite this, we believe the observed patterns reflect natural conditions because the feeding rates for individuals and groups (mean of ca. 5 clams/fish when alone and 10 clams/fish when foraging together) are in line with previous publications using wild-caught Florida Pompano. In our experiment, the number of clams consumed by individual maricultured fish when in groups was ca. 2 times greater than that for Lindquist and Manning [32] who examined the effect of turbidity on Florida Pompano foraging on coquina clams. Another experiment assessing potential preference for clams with or without hydroids present resulted in an average consumption of 7 clams within 5 min [17]. This would suggest a much higher feeding rate by wild-caught Florida Pompano (ca. 84 clams/hr); however, there was a fundamental difference in experimental procedures. Manning and Lindquist [17] added the clams to the tanks after the pompano were present. Since Florida Pompano are sight feeders and potentially perceived the prey as being available for a short time period, they likely went after the clams more readily. Captive Florida Pompano feeding rates appear to be context-dependent but the maricultured fish used in our experimental trials consumed an intermediate amount of clams. Furthermore, maricultured fish responded to visual cues, participated in foraging activities, and exhibited feeding rates similar to wild-caught Florida Pompano that were previously held at DISL (Schrandt, pers. obs.). Overall, we believe the patterns observed here (i.e. increased foraging when in groups) may generally reflect feeding patterns in wild populations since the maricultured fish fed, responded to visual cues, and had feeding rates within the range of published rates for wild-caught Florida Pompano of similar size.

## Conclusions

In concert, our results suggest that groups facilitate foraging even after locating a prey patch and that juvenile Florida Pompano are more comfortable and successful foraging in groups. This provides new information on the foraging ecology of Florida Pompano, with both ecological and economic implications. No previous studies have addressed group-feeding behaviors of Florida Pompano foraging on the main contributor to their natural diet. Ultimately, schooling may facilitate juvenile Florida Pompano feeding activities along the sandy beach habitat, an area where presumably (1) feeding is difficult because of the dynamic environment and (2) little protection is available from predators. Furthermore, our results are applicable to Florida Pompano aquaculture. Because foraging (and hence growth) is facilitated by groups, Florida Pompano should be reared with conspecifics, preferably of similar size to potentially reduce the dominance effect we observed. Periodically size-separating fish during the rearing period could lead to more efficient growth and harvest of Florida Pompano.
